# Higher global diet quality score is related to lower prevalence of depression and poor quality of life among adolescent girls

**DOI:** 10.1186/s12888-023-05313-7

**Published:** 2023-11-28

**Authors:** Sara Beigrezaei, Zahra Darabi, Ian G. Davies, Mohsen Mazidi, Majid Ghayour-Mobarhan, Sayyed Saeid Khayyatzadeh

**Affiliations:** 1 Research Center for Food Hygiene and Safety, School of Public Health, Shahid Sadoughi University of Medical Sciences, Yazd, Iran; 2 Department of Nutrition, School of Public Health, Shahid Sadoughi University of Medical Sciences, Yazd, Iran; 3 Research Institute of Sport and Exercise Science, Liverpool John Moores University, Liverpool, UK; 4 Medical Research Council Population Health Research Unit, University of Oxford, Oxford, UK; 5 Nuffield Department of Population Health, Clinical Trial Service Unit and Epidemiological Studies Unit (CTSU), University of Oxford, Oxford, UK; 6 Department of Twin Research and Genetic Epidemiology, King’s College London, South Wing St Thomas’, London, UK; 7 International UNESCO center for Health-Related Basic Sciences and Human Nutrition, Mashhad University of Medical Sciences, Mashhad, Iran; 8 Nutrition and Food Security Research Center, Shahid Sadoughi University of Medical Sciences, Shohadaye gomnam BLD. ALEM square, Yazd, Iran

**Keywords:** Depression, Quality of life, Global dietary quality score, GDQS, Cross-sectional, Adolescents

## Abstract

**Background:**

Adolescence is a key time for the development of depression symptoms and the diet quality may be associated with mental health conditions. The present study examined the association between depression and quality of life (QoL) and the global diet quality score (GDQS) as a simple and standardized metric diet quality in Iranian adolescents.

**Methods:**

This cross-sectional study was conducted on 733 adolescent girls recruited using a random cluster sampling method. A 147-item food frequency questionnaire (FFQ) was used for dietary intake assessment. The GDQS is gained by summing points of all the 25 food groups, ranged from 0 to 49. Depression symptoms were assessed using a Persian version of the Beck Depression Inventory (BDI). For assessment of health-related QoL, the Short Form 12 Survey–version 2 (SF-12v2) questionnaire was employed. Multivariable logistic regression examined the association of depression and QoL with GDQS in crude and adjusted models.

**Results:**

Adolescent girls in the highest tertile of GDQS score compared with the lowest tertile had a 41% lower odds of depressive symptoms (OR: 0.59; 95% CI: 0.39–0.90, P = 0.01). The participants in the third tertile of GDQS score had lower odds of poor QoL compared with the first tertile (OR: 0.56; 95% CI: 0.37–0.85, P < 0.01). These associations remained significant (both P = 0.01) after adjustment for age, energy intake, body mass index (BMI), physical activity, and menstruation (depressive symptoms: OR: 0.59; 95% CI: 0.38–0.92; QoL: OR: 0.59; 95% CI: 0.38–0.91, P = 0.01).

**Conclusion:**

We found that adolescent girls with a higher score of the GDQS had lower odds of depression and poor QoL Prospective and interventional investigations are needed to reach a clear vision.

**Supplementary Information:**

The online version contains supplementary material available at 10.1186/s12888-023-05313-7.

## Background

Depression is a common mental disorder worldwide [1], a major cause of disability, and is the main contributor to the global burden of disease [2]. Evidence has reported that women are more affected by depression than men [3]. Depression raises the risk of chronic diseases such as type 2 diabetes mellitus and cardiovascular diseases [2]; moreover, it is the main cause of death from suicide in the world [4]. Depression generally first emerges throughout adolescence and affects 8 to 20% of adolescents < 18 years worldwide [5]. In addition, depression can negatively affect the psychological and physical health of adolescents and result in some behavioral and social consequences including decreased quality of life (QoL) [6, 7]. QoL is a multi-dimensional concept of an individual’s overall well-being situation in association with the value, cultural, social, and environmental circumstances in which they live [8].

As such, the identification and improvement of modifiable risk factors for adolescent depression can benefit their QoL [9]. Poor-quality diet is a major cause of unfavorable health outcomes [10, 11]. Depression has been linked to diet quality and a number of studies show associations between lower diet quality and higher depression risk in adolescents [12, 13]. For example, a cross-sectional study on adolescents aged 11–14 years reported an association between higher intake of unhealthy food items and symptoms of mental health [12]. Moreover, Jacka et al. [14] found that 10–14-year old adolescents with adherence to a less healthy diet had increased odds of depressive symptoms. Jacka et al. [15], in a separate study, also showed a dose response relationship between a higher healthy diet quality score and higher scores of health-related QoL in adolescents aged 11–18 years. However, McMartin et al. [16] did not reveal a link between diet quality and depression and anxiety in children aged 10–11 years.

Recently, the global dietary quality score (GDQS) was developed as a new tool to comprehensively evaluate the quality of diet [17].

The GDQS metric consists of 25 food groups that are based on current nutrition science and epidemiologic literature and that have a significant impact on nutrient intake and/or non-communicable disease (NCD) risk globally [17–19]. The GDQS sub metrics can be further subdivided to give more detailed information about the contribution of smaller sets of food groups or individual food groups to diet quality in populations [17]. Some of these food groups include whole/ refined grains, Nuts and seeds, deep orange tubers, deep orange vegetables and fruits, meats, dairies, etc. [17]. Though several metrics for diet quality have been developed worldwide, there is still not a validated, relatively simple, and widely used metric to assess the quality of diet in population-based surveys [17]. The GDQS provides an appropriate simple and standardized metric for the population-based surveys of diet quality [17]. Evidence from prospective studies is consistent that adherence to this diet quality index is associated with a lower risk of diabetes and obesity and less weight gain in US women [20, 21].

To our knowledge, this is the first study to assess the association between mental health conditions and diet quality assessed by the GDQS, as a global score system. Therefore, in the present study, we aimed to evaluate diet quality in association with depression and QoL in adolescent girls.

## Materials and methods

### Study population

We used a random cluster sampling method to select 1026 adolescent girls (aged 12–18 years) from several schools in Mashhad and Sabzevar, northeastern Iran, for this cross-sectional study. In the first step, the 8 educational districts of Mashhad and Sabzevar were allocated to 4 clusters because of socioeconomic similarity. Then, one district from each cluster was randomly select. In the next step, two high schools will randomly select from each selected district. We excluded 38 subjects for various reasons and 255 subjects for under-reporting or over reporting their energy intake. Our final analysis included 733 adolescent girls.

Adolescent girls between the ages of 12 and 18 were included in this study. Participants with history of chronic diseases such as colitis, diabetes, cardiovascular disease (CVD), cancer, and hepatitis, individuals taking anti-inflammatory, antidepressant, anti-diabetic, or anti-obesity.

drugs as well as those on vitamin D, calcium supplementation, or hormone therapy within the last six months were not included. All adolescents and their parents provided complete written informed consent/assent before participating in the study. The study was approved by the ethic committee of Mashhad University of Medical Sciences (MUMS) (code number: 931,188).

### Dietary assessment

A 147-item FFQ assessed dietary intakes by face-to-face interview, regarding daily, weekly, monthly and yearly intake, over the last year. Validity and reliability of this questionnaire has been previously reported [22, 23]. The reported portion sizes were converted to grams using household measures, and the energy and nutrient intakes were calculated using the Nutritionist IV software [10].

### GDQS calculation

The GDQS metric is composed of 25 food groups (Supplementary Table 1). Points are attributed based on three or four categories of consumed amounts (defined in g/d) specific to each group. Supplementary Table 1 described the scoring of 25 food groups. This was broken down into 16 healthy food groups, of which higher intake received higher points; seven unhealthy food groups, receiving more points for lower intake, and ultimately two food groups categorized as unhealthy when consumed in excessive amounts (more points for lower intake). The GDQS is scored by summing points of all 25 food groups, ranging from 0 to 49 [17].

### Assessment of depression

Depression symptoms were assessed using a Persian version of the Beck Depression Inventory (BDI). The validity and reliability of this questionnaire was confirmed in previous studies [24, 25]. This questionnaire included 21 items that evaluated various symptoms of depression; feelings of guilt, hopelessness, and sadness; crying, sleep disturbance, fear and loss of appetite over the past 2 weeks. The range of scores for the BDI was between 0 and 63. If the BDI score was < 16, the individuals were considered as not depressed, and if this score was ≥ 16, they were categorized as depressed [26].

### Quality of life assessment

For assessment of health-related QoL, the SF-12v2 questionnaire was used. This questionnaire was a short form of the SF-36 questionnaire and a modified version of the SF-12v1 [27]. The validity and reliability of the questionnaire were confirmed in a previous Iranian study [28]. The questionnaire includes 12 items that evaluated 8 domains of health including physical function, physical role (physical health problems), bodily pain, general health, vitality, social functioning, emotional role (emotional problems), and mental health. The QoL scores range from 0 to 100. We used a median cut-point for the definition of poor QoL; therefore, the subjects were categorized as having poor QoL if their score was < 43.

### Demographic data collection and anthropometric measurements

General demographic data were gathered by experienced interviewers. This data included age, smoking status, menstruation status, medical history, supplement use, psychological.

treatment and chronic diseases. Anthropometric measurements were performed by trained investigators using standard protocols (including weight, height and waist circumferences (WC)).

Weight was measured using a digital scale with an accuracy of 100 g, while participants were without shoes in a minimal clothing state. Height (without shoes) was measured using a stadiometer with an accuracy of 0.1 cm. Body mass index (BMI) was calculated as weight (kg).

divided by height squared (m^2^). In addition, WC was measured at the narrowest level between the costal margin and iliac crest during minimal respiration in a standing position. Physical activity level was assessed using the Persian-translated modifiable activity questionnaire (MAQ) with high reliability and moderate validity [29]. Data on the time and frequency of light, moderate, high, and very hard intensity activities were obtained according to the list of common activities of daily life over the past year, and these activity data were transformed into metabolic equivalent-hours/week (Met/hours/week; 1 MET = 3.5 mL kg-1 min-1 of O2 consumption).

### Statistical analysis

The participants were classified into tertiles of GDQS. General characteristics, biochemical parameters and nutrient intake across tertiles of GDQS were expressed as means ± standard deviations (SDs) for continuous variables, and as frequencies and percentages for categorical variables. To examine differences between tertiles, one-way-ANOVA and chi-square tests were used for continuous and categorical variables, respectively. To investigate the relationship between GDQS with depression and QoL, multivariate and linear regression were conducted with crude and adjusted models (Model 1: adjusted for age and energy intake; Model 2: additionally, adjusted for BMI percentile; Model 3: additionally, adjusted for physical activity, menstruation). All statistical analyses were conducted using SPSS version 21. P-values < 0.05 were considered statistically significant.

## Results

### General characteristics study participants

Current cross-sectional study indicated that higher adherence to GDQS inversely associated to poor QoL and depression prevalence.

The mean ± SD age of the participants was 14.5 ± 1.53 years. The prevalence of depression and poor QoL were 24% and 49%, respectively. General characteristics and anthropometric variables of the participants across tertiles of GDQS score were presented in Table 1. The participants in the lowest tertile of GDQS score had significantly lower percentile BMI compared to participants in the highest tertile of GDQS score (P < 0.01). Age, waist circumference, waist-to-hip ratio, physical activity and menstruation were not different among tertiles of GDQS score.

**Table 1:** General characteristics of study participants by tertile of GDQS

	T1	T2	T3	P value*
Age(year)	14.70 ± 1.608	14.40 ± 1.524	14.53 ± 1.467	0.09
Percentile BMI (kg/m2 )	42.00 ± 28.65310	49.36 ± 28.48770	50.76 ± 29.36819	< 0.01
Waist circumference (cm)	69.47 ± 8.121	70.47 ± 9.460	71.62 ± 9.221	0.07
Waist-to-hip ratio	0.76 ± 0.05294	0.76 ± 0.06753	0.76 ± 0.05575	0.79
Physical activity (MET h/week)	45.05 ± 2.85	45.39 ± 3.63701	45.67 ± 3.60948	0.21
Menstruation (yes) n (%)	170 (91.4)	322 (89.2)	170 (93.4)	0.26
Quality of life score	41.30 ± 7.84	41.77 ± 8.10	43.47 ± 7.75	0.02
Depression score	11.56 ± 8.88	11.48 ± 9.50	9.16 ± 9.03	0.01

BMI: Body mass index; GDQS: Global Diet Quality Score

Values are means ± SD and number (prevalence percent) for quantitative and qualitative variables, respectively.

*Obtained from one way Anova for continuous variables and Chi-squared test for categorical variables

### Dietary intake of study participants

Dietary intake of study participants across tertiles of GDQS score are shown in Table 2. Participants in the highest tertile of GDQS score compared to the participants who were in the lowest tertile had higher intake of energy, percent of protein, carbohydrate and fat. Intake of vitamins A, C, B6 and calcium were significantly higher in participants in the third tertile of the GDQS score compared to first tertile (per 1000 kcal). As expected, participants in the lowest tertile of the GDQS score had lower intake of citrus fruits, dark green leafy vegetables, cruciferous vegetables, deep orange vegetables, legumes, nuts and seeds, liquid oils, fish and shellfish, poultry and game meat, egg, and total dairy compared with the highest tertile.

**Table 2:** Energy and dietary intakes of study participants by tertile of GDSQ

	T1	T2	T3	P-value*
Citrus fruits (g)	24.12 ± 53.99	28.98 ± 32.95	38.77 ± 41.17	< 0.01
Other fruits (g)	134.96 ± 132.69	179.16 ± 172.79	231.53 ± 168.67	< 0.001
Dark green leafy vegetables (g)	16.91 ± 18.53	36.87 ± 40.79	58.44 ± 43.06	< 0.001
Cruciferous vegetables (g)	6.94 ± 17.74	11.21 ± 18.10	17.06 ± 19.78	< 0.001
Deep orange vegetables (g)	0.27 ± 1.11	0.54 ± 1.94	0.47 ± 1.68	0.15
Other vegetables (g)	103.35 ± 112.49	145.00 ± 156.23	179.14 ± 141.59	< 0.001
Legumes (g)	49.88 ± 51.51	71.36 ± 50.51	92.76 ± 71.34	< 0.001
Deep orange tubers (g)	40.32 ± 44.86	37.07 ± 30.91	44.69 ± 36.42	0.08
Nuts and seeds (g)	10.18 ± 26.69	15.43 ± 33.29	24.30 ± 27.63	< 0.001
Whole grains (g)	60.31 ± 116.32	57.37 ± 106.03	51.15 ± 79.78	0.59
Liquid oils (g)	4.27 ± 7.41	5.75 ± 7.33	8.13 ± 8.81	< 0.001
Fish and shellfish (g)	5.74 ± 8.97	7.57 ± 9.77	12.17 ± 21.22	< 0.001
Poultry and game meat (g)	18.89 ± 22.05	23.93 ± 22.16	30.90 ± 27.05	< 0.001
Low fat dairy (g)	180.17 ± 173.82	242.67 ± 195.04	309.57 ± 240.83	< 0.001
Eggs (g)	15.31 ± 16.29	20.96 ± 20.99	25.43 ± 24.96	< 0.001
High fat dairy (g)	143.65 ± 153.49	190.44 ± 180.66	222.21 ± 164.88	< 0.001
Red meat (g)	11.45 ± 17.24	13.78 ± 17.02	15.30 ± 13.37	0.02
Processed meat (g)	7.31 ± 9.74	7.81 ± 10.11	7.68 ± 10.23	0.85
Refined grains and baked goods (g)	423.02 ± 236.07	413.33 ± 224.04	458.13 ± 228.43	0.08
Sweets and ice cream (g)	50.45 ± 37.63	49.28 ± 42.25	45.79 ± 49.08	0.45
Sugar-sweetened beverages (g)	80.76 ± 142.33	51.69 ± 89.52	39.67 ± 60.92	< 0.001
Juice (g)	54.19 ± 97.95	50.85 ± 92.62	71.87 ± 194.00	0.19
White roots and tubers (g)	8.29 ± 15.712	9.29 ± 12.90	1.63 ± 26.17	0.13
Purchased deep fried foods (g)	30.65 ± 31.04	33.95 ± 32.11	33.43 ± 27.52	0.42
Energy intake (Kcal)	2453.92 ± 832.39	2660.46 ± 765.80	3033.56 ± 786.70	< 0.001
Carbohydrate (% energy)	56.32 ± 8.08	54.17 ± 7.01	53.89 ± 6.43	< 0.001
Protein (% energy)	12.87 ± 2.24	13.86 ± 2.13	14.22 ± 2.07	< 0.001
Fat (% energy)	33.04 ± 8.66	34.29 ± 7.42	34.21 ± 6.77	0.12
Cholesterol (mg/1000 Kcal)	78.92 ± 47.03	93.88 ± 47.58	95.91 ± 39.43	< 0.001
Saturated fatty acid (gr/1000 Kcal)	10.55 ± 3.36	11.66 ± 3.32	11.52 ± 3.28	< 0.001
Monounsaturated fatty acid (gr/1000 Kcal)	12.05 ± 0.95	12.20 ± 3.26	12.13 ± 3.08	0.88
Polyunsaturated fatty acid (gr/1000 Kcal)	8.41 ± 3.61	8.41 ± 3.04	8.31 ± 2.76	0.92
Calcium (mg/1000 Kcal)	387.75 ± 125.18	436.79 ± 139.06	456.74 ± 130.93	< 0.001
Iron (mg/1000 Kcal)	7.41 ± 1.62	7.29 ± 1.41	7.43 ± 1.33	0.55
Vitamin A (mcg/1000 Kcal)	192.01 ± 371.85	219.71 ± 114.60	262.46 ± 119.93	< 0.01
Vitamin C (mg/1000 Kcal)	30.15 ± 20.39	35.83 ± 19.33	41.21 ± 20.41	< 0.001
Folate (mcg/1000 Kcal)	226.80 ± 55.74	226.96 ± 45.36	231.20 ± 45.40	0.53
Vitamin B6 (mg/1000 Kcal)	0.67 ± 0.13	0.72 ± 0.12	0.73 ± 0.11	< 0.001
Vitamin B12 (mcg/1000 Kcal)	1.55 ± 3.79	1.57 ± 0.75	1.65 ± 0.72	0.88

GDQS: Global Diet Quality Score

Values are means ± SDs

*Obtained from One way Anova

### Association between GDQS score and depression and poor QoL

Multi-variable adjusted odds ratios (ORs) for depression and poor QoL categories across tertiles of GDQS score are represented in Table 3. Adolescent girls in the highest tertile of GDQS score compared with the lowest tertile had a 41% lower probability of depressive symptoms (OR: 0.59; 95% CI: 0.39–0.90, P = 0.01). The participants in the third tertile of GDQS score had lower odds of poor QoL compared with the subjects in the first tertile (OR: 0.56; 95% CI: 0.37–0.85, P < 0.01). These associations remained significant after adjustment for age, energy intake, BMI percentile, physical activity and menstruation (Depression: OR: 0.59; 95% CI: 0.38–0.92; QoL: OR: 0.59; 95% CI: 0.38–0.91, P ≤ 0.01 for both).

**Table 3:** Multivariable-adjusted odds ratio (OR) of the associations between GDQS with poor quality of life and depression

	T1 Reference	T2 OR (95%CI)	T3 OR (95%CI)	P value¹	P trend
Poor quality of life					
Crude	1.00	0.97 (0.68–1.39)	0.56(0.37–0.85)	< 0.01	< 0.01
Model1	1.00	0.98(0.68–1.41)	0.56(0.36–0.86)	< 0.01	< 0.01
Model2	1.00	1.01(0.70–1.45)	0.58(0.37–0.89)	0.01	0.01
Model3	1.00	1.04(0.72–1.51)	0.59(0.38–0.91)	0.01	0.01
Depression					
Crude	1.00	0.65(0.43–0.98)	0.59(0.39–0.90)	0.01	0.01
Model1	1.00	0.65(0.43–0.98)	0.59(0.39–0.91)	0.01	0.01
Model2	1.00	0.66(0.43–0.99)	0.60(0.39–0.93)	0.02	0.01
Model3	1.00	0.65 (0.42–0.98)	0.59(0.38–0.92)	0.01	0.01

GDQS: Global Diet Quality Score; OR: odds ratio; CI: confidence interval.

¹Last tertile compared to first teretile.

Model 1: Adjusted for age and energy intake.

Model 2: Additionally, adjusted for BMI percentile.

Model 3: Additionally, adjusted for physical activity, menstruation.

The association between Score of depression and quality of life with GDQS were presented in Table 4. There is a inverse relationship between GDQS with depression score (β= -0.010) and a positive relationship with quality of life score (β = 0.095) in crude model.

**Table 4:** The association between Score of depression and quality of life with GDQS

Food intake	Score of Quality of life		Score of Depression	
	ʙ	P value	ʙ	P value¹
**GDQS**				
Crude	-0.1	< 0.01	0.095	0.1
Model1	-0.11	0.004	0.106	< 0.01
Model2	-0.11	0.005	0.106	< 0.01
Model3	-0.11	0.004	0.101	0.01

GDQS: Global Diet Quality Score

^1^Obtained from linear regression

Model 1: Adjusted for age and energy intake

Model 2: Additionally, adjusted for BMI percentile

Model 3: Additionally, adjusted for physical activity, menstruation

## Discussion

In the present cross-sectional study, we found that adolescent girls with a higher score of the GDQS had a lower risk of depression and poor QoL. This favorable association remained in multivariate models after controlling for some lifestyle-related variables. To our knowledge, this is the first study to investigate GDQS and risk of depression and poor QoL.

In line with our findings, other studies have reported significant relationships between depression and diet quality. Gibson-Smith et al. [30] showed the Mediterranean diet score and the Alternative healthy eating index (AHEI), as measures of diet quality, are lower in adults with depression. A prospective study in midaged females reported that long-term maintenance of good diet quality may be related to reduced depression risk (9). Moreover, a systematic review of observational studies showed a link between a diet rich in healthy foods and lower levels of depression in adolescents (31). Whereas, Winpenny et al. [32] reported no prospective or cross-sectionally associations between diet quality, fruit and vegetable intake, or fish intake with depressive symptoms in adolescence. Some studies were conducted in IRAN [33, 34]. Results of a cross-sectional study, showed that moderate adherence to the Dietary Approaches to Stop Hypertension (DASH) dietary pattern was inversely associated with depression in Iranian adults. The DASH-style diet is a healthy dietary pattern that consists of fruits, vegetables, low-fat dairy, and plant proteins from nuts and legumes, and that limits red meat, sweets, and sugar-sweetened beverages (33). Another study has suggested that Iranian adolescent females who consumed a pro-inflammatory diet, as measured by higher dietary inflammatory index (DII) scores, were more likely to have depressive symptoms (34).

In our study, GDQS was associated with adolescent QoL cross-sectionally. Jacka et al. [15] in both longitudinal and cross-sectional studies showed the diet quality of Australian adolescents was related to their mental health according to scores on the emotional subscale of the Pediatric Quality of Life Inventory. In addition, Esteban-Gonzalo et al. [35], from cross-sectional analysis, revealed that high-quality diet (using the Mediterranean diet) adherence was directly associated with better health-related –QoL.

There are many nutritional components of a diet with high GDQS that may have a direct effect on various mechanisms that underpin depression and QoL, including the immune system and oxidative stress [31]. Compelling evidence from recent meta-analyses has demonstrated that a more inflammatory diet increases the risk of depression [36].

Higher scores of GDQS are related to higher adherence to a healthy dietary pattern containing high amounts of fish, fruits, vegetables, whole grains, low-fat dairy, nuts, and legumes. Some of these healthy foods are of interest due to their role in reducing inflammation [37, 38]. Whole grains as a main source of fiber is associated with increasing adiponectin and decreasing inflammatory markers [39]. Moreover, dietary phytochemicals like polyphenols have anti-inflammatory effects on neuropsychiatric systems [37]. High concentrations of omega-3 polyunsaturated fatty acids in marine foods, due to their anti-inflammatory properties, delay the beginning of cytokine-induced depression and play a key role in the regulation of emotions [40, 41].

In addition, the high content of antioxidants such as carotenoids, vitamin E, and vitamin C in fruits and vegetables as part of the GDQS have protective impacts against depression risk [42, 43]. The protective effect of antioxidants can be attributed to mechanisms that repair neuronal damage, especially the hippocampus neurons, which are related to depression [43]. The hypothalamus- pituitary-adrenal (HPA) axis through regulating glucocorticoid production contributes to the pathophysiology of neuropsychiatric disorders [44]. Evidence suggests people living with depression have disturbances to the HPA axis and excessive cortisol production [44]. Clinical trials with nutrient interventions, such as vitamin C, have revealed a reduction in the reactivity of cortisol to acute physiological stress in healthy adults [45]. Additionally, interventions using polyphenol-rich foods have shown a remarkably decrease in cortisol levels [46, 47]. Some studies reported that omega-3 fatty acids could improve cortisol levels in healthy and depressive adults, as well [48].

On the other hand, high intake of processed foods is associated with inflammation, which can contribute in the pathogenesis of depression [49, 50]. A cross-sectional study established that the Western-type diet, containing high amounts of processed meat, refined grains, high-fat and high-sugar foods was linked to greater levels of low-grade inflammation [51] and the subsequent atrophy of the brain, which was directly related to depression [52]. Moreover, evidence has demonstrated that low-grade inflammation is becoming growingly common in adolescents [15]. In addition, it is demonstrated that dietary patterns rich in sweets and soft drinks were associated with an increased depression risk due to the alteration of endorphin levels and oxidative stress [53].

Although it is thought that depression can cause adherence to an unhealthy diet, some longitudinal studies have proposed that this direction of causality is a less likely justification for long-term associations [54]. Previous evidence proposed that sugary and sweetened food items may alleviate the distress of people with mood disturbance in short term. This relationship can have calmative impacts in the short term.

To the best of our knowledge, this is the first study to explore associations between GDQS and depression and QoL in adolescents. Our findings provide the basis for future investigation to identify any causal link between GDQS and depression and QoL. The large sample size and a wide range of covariates involved in the analyses add strength to our study. However, there are some limitations regarding our findings that should be considered. The direction of the associations cannot be determined in cross-sectional studies. In addition, evaluating dietary intakes with the FFQ is prone to misreporting (over/underestimation) and misclassification. This study was entirely conducted on adolescent girls, limiting the generalizability of our findings to populations beyond this sample.

## Conclusion

In summary, our findings demonstrated that greater GDQS is associated with lower depression prevalence and poor QoL in adolescent girls. Children and their parents should be informed which dietary intakes of high sources of antioxidant and fiber and lower consumption of sugar and saturated fatty acids improve psychological health. To confirm these findings, further research needs to be conducted in different areas, including longitudinal studies with larger sample sizes.

### Electronic supplementary material

Below is the link to the electronic supplementary material.

Supplementary Material 1

## Data Availability

The data and materials of the current study is available from the corresponding author on reasonable request.
