# Supplementary material for: Higher global diet quality score is related to lower prevalence of depression and poor quality of life among adolescent girls
Source: BMC Psychiatry. 2023 Nov 28;23:886. doi: 10.1186/s12888-023-05313-7 (PMC10683251; doi:10.1186/s12888-023-05313-7)
Supplement: Supplementary file 1 — Supplementary Material 1 [file 12888_2023_5313_MOESM1_ESM.docx]

| Supplementary Table 1. GDQS and GDQS Sub-Metric Food Groups and Scoring | | | | | | | | | | | |
| --- | --- | --- | --- | --- | --- | --- | --- | --- | --- | --- | --- |
| Inclusion in Metrics | Scoring Classification | Food Group | Categories of Consumed Amounts (g/day) | | | | Points Assigned | | | | |
|  |  |  | Low | Middle | High | Very High | Low | | Middle | High | Very High |
| GDQS and GDQS+ | Healthy | Citrus fruits | <24 | 24-69 | >69 |  | 0 | | 1 | 2 |  |
|  |  | Deep orange fruits | <25 | 25-123 | >123 |  | 0 | | 1 | 2 |  |
|  |  | Other fruits | <27 | 27-107 | >107 |  | 0 | | 1 | 2 |  |
|  |  | Dark green leafy vegetables | <13 | 13-37 | >37 |  | 0 | | 2 | 4 |  |
|  |  | Cruciferous vegetables | <13 | 13-36 | >36 |  | 0 | | 0.25 | 0.5 |  |
|  |  | Deep orange vegetables | <9 | 9-45 | >45 |  | 0 | | 0.25 | 0.5 |  |
|  |  | Other vegetables | <23 | 23-114 | >114 |  | 0 | | 0.25 | 0.5 |  |
|  |  | Legumes | <9 | 9-42 | >42 |  | 0 | | 2 | 4 |  |
|  |  | Deep orange tubers | <12 | 12-63 | >63 |  | 0 | | 0.25 | 0.5 |  |
|  |  | Nuts and seeds | <7 | 7-13 | >13 |  | 0 | | 2 | 4 |  |
|  |  | Whole grains | <8 | 8-13 | >13 |  | 0 | | 1 | 2 |  |
|  |  | Liquid oils | <2 | 2-7.5 | >7.5 |  | 0 | | 1 | 2 |  |
|  |  | Fish and shellfish | <14 | 14-71 | >71 |  | 0 | | 1 | 2 |  |
|  |  | Poultry and game meat | <16 | 16-44 | >44 |  | 0 | | 1 | 2 |  |
|  |  | Low-fat dairy | <33 | 33-132 | >132 |  | 0 | | 1 | 2 |  |
|  |  | Eggs | <6 | 6-32 | >32 |  | 0 | | 1 | 2 |  |
| GDQS and GDQS– | Unhealthy in excessive amounts | High-fat dairy | <35 | 35-142 | 142-734 | >734 | 0 | | 1 | 2 | 0 |
|  |  | Red meat | <9 | 9-46 | >46 |  | 0 | | 1 | 0 |  |
|  | Unhealthy | Processed meat | <9 | 9-30 | >30 |  | 2 | 1 | | 0 |  |
|  |  | Refined grains and baked goods | <7 | 7-33 | >33 |  | 2 | | 1 | 0 |  |
|  |  | Sweets and ice cream | <13 | 13-37 | >37 |  | 2 | | 1 | 0 |  |
|  |  | Sugar-sweetened beverages | <57 | 57-180 | >180 |  | 2 | | 1 | 0 |  |
|  |  | Juice | <36 | 36-144 | >144 |  | 2 | | 1 | 0 |  |
|  |  | White roots and tubers | <27 | 27-107 | >107 |  | 2 | | 1 | 0 |  |
|  |  | Purchased deep fried foods | <9 | 9-45 | >45 |  | 2 | | 1 | 0 |  |
